# Optimization of Extraction Process for Flavonoids from *Sonchus oleraceus* L. and Evaluation of Anti-Inflammatory Activity of Luteoloside

**DOI:** 10.3390/molecules31071105

**Published:** 2026-03-27

**Authors:** Ke Sheng, Junyao You, Shuai Tian, Yaling Lu, Jiamin Wu, Jianping Zhang

**Affiliations:** 1 Xinjiang Production and Construction Corps Key Laboratory of Protection and Utilization of Biological Resources in Tarim Basin, College of Life Science and Technology, Tarim University, Alar 843300, China; 15954101003@163.com (K.S.); you001120@163.com (J.Y.); 18867246482@163.com (S.T.); w18628469320@163.com (J.W.); 2 College of Chemistry and Chemical Engineering, Tarim University, Alar 843300, China; skyling019@163.com

**Keywords:** Xinjiang *Sonchus oleraceus* L., flavonoids, response surface methodology, luteoloside, anti-inflammatory activity

## Abstract

*Sonchus oleraceus* L., a member of the Asteraceae family native to Eurasia, is a herbaceous plant whose young stems and leaves are consumed globally as a medicinal and edible wild vegetable; it is rich in flavonoids and exhibits various pharmacological activities, including anti-inflammatory and anti-tumor effects. This study optimized the extraction process of flavonoids from Xinjiang *S. oleraceus* using response surface methodology and evaluated the anti-inflammatory activity of luteoloside in vitro. Based on single-factor experiments and Box–Behnken design, the effects of ethanol concentration, extraction time, solid-to-liquid ratio, and extraction temperature on flavonoid yield were investigated. The optimal extraction conditions were determined as ethanol concentration 62%, extraction time 30 min, solid-to-liquid ratio 1:91 g/mL, and extraction temperature 64 °C, with a flavonoid yield of 21.64 mg/g. After purification via polyamide column chromatography, the luteoloside content was determined by HPLC to be 44.06 μg/g. Cytotoxicity assays revealed that a luteoloside concentration of 100 μmol/L reduced the viability of *Oryctolagus cuniculus* colon epithelial cells to approximately 80%. ELISA results demonstrated that luteoloside significantly inhibited the release of pro-inflammatory factors, including TNF-α, while promoting the expression of the anti-inflammatory factor IL-10. These findings indicate that luteoloside effectively alleviates LPS-induced cellular inflammation.

## 1. Introduction

*Sonchus oleraceus* L. is an annual or biennial herbaceous plant of the Asteraceae family, typically growing 20–40 cm tall with a spreading, clump-branched stem and distinctive leaves that are linear–lanceolate to elliptic–lanceolate in shape, often featuring irregular coarse serrations or even pinnate shallow to deep lobes, and an auriculate, stem-clasping base [1]. Native to Europe and the Mediterranean region, the young stems and leaves of *S. oleraceus* are harvested as wild vegetables or processed into dietary supplements in many parts of Asia, Europe, and Oceania [2]. For instance, in southern and central–western Brazil, its leaves—locally known as serralha, chicória-brava, or serralheira [3]—are valued for their spinach-like bitterness and are commonly used in salads [4]. Flavonoids are among the major functional components in *S. oleraceus*, and numerous studies have revealed that they contribute significantly to the plant’s diverse bioactivities [5]. Extensive in vitro, in vivo, and emerging clinical research has demonstrated that *S. oleraceus* extracts exhibit notable pharmacological effects, including anti-inflammatory [6], analgesic [7], anxiolytic [8], antitumor [9], and anti-aging properties [10], providing a solid scientific foundation for its further development in pharmaceutical and functional food applications.

Flavonoids are a class of natural polyphenolic compounds with diverse biological activities, characterized by a core C_6_-C_3_-C_6_ carbon skeleton formed by two aromatic rings (A-ring and B-ring) linked through a three-carbon chain [11]. Widely distributed throughout the plant kingdom, these compounds are important secondary metabolites commonly found in various plant tissues such as roots, stems, leaves, flowers, fruits, and seeds, where they play crucial roles in pest resistance and growth regulation [11]. Numerous studies have confirmed their multiple bioactivities, including antibacterial, antiviral, antitumor, antioxidant, and anti-inflammatory effects, demonstrating broad application prospects in the development of functional foods, pharmaceuticals, and cosmetics [12]. Regarding extraction techniques, ultrasound-assisted solvent extraction is currently the mainstream method for obtaining plant-derived flavonoids, leveraging the cavitation, mechanical vibration, and thermal effects generated by ultrasound to effectively disrupt plant cell walls, accelerate solvent penetration, and enhance the dissolution of target compounds, thereby significantly reducing extraction time and substantially improving yield [13]. To systematically optimize such extraction processes, Response Surface Methodology (RSM) has become the preferred experimental design and optimization tool, capable of precisely analyzing the influence of key factors—such as extraction temperature, time, solvent concentration, and solid-to-liquid ratio—and their complex interactions on extraction yield through well-fitted mathematical models [14]. Compared to the traditional “one-factor-at-a-time” approach, RSM offers distinct advantages including shorter experimental cycles, higher regression accuracy, and superior predictive performance, making it widely adopted for process parameter optimization in fields such as agriculture, biology, food science, and chemical engineering [15].

Luteoloside, also known as luteolin-7-O-glucoside, is a widely distributed flavonoid glycoside in nature, significantly present in various medicinal and edible plants as one of their key active components [16]. The ortho-dihydroxyl groups on its core structure enable luteoloside to effectively chelate metal ions and stabilize free radicals through electron transfer, thereby exhibiting potent antioxidant activity and making it an effective agent for scavenging excess free radicals in the body [17]. Based on this chemical framework, modern pharmacological studies have revealed the compound’s diverse properties: beyond its well-recognized anti-inflammatory [18], antibacterial [19], and antioxidant [20] effects, extensive in vitro and animal model research further confirms its potential in antitumor applications, with mechanisms that may include inducing tumor cell apoptosis [21] and inhibiting proliferation and metastasis [22]. Additionally, studies have reported its bioactivities in cardiovascular protection [23], neuroprotection [24], and antiviral [25] effects, establishing luteoloside as a promising candidate molecule in natural product research and new drug development.

This study utilized Xinjiang *S. oleraceus* as the experimental subject, aiming to systematically investigate the extraction, purification, and anti-inflammatory efficacy of its active components. First, ethanol was employed as the solvent, combined with ultrasound-assisted extraction technology, to efficiently extract total flavonoids from *S. oleraceus*. Subsequently, by integrating single-factor experiments with response surface methodology, key parameters in the extraction process—such as solid-to-liquid ratio, ethanol concentration, extraction temperature, and time—were systematically optimized. After obtaining the crude total flavonoid extract, sequential purification was performed using D101 macroporous resin and polyamide column chromatography to directionally enrich the key active component—luteoloside. During the purification process, high-performance liquid chromatography (HPLC) was utilized for the quantitative analysis of luteoloside content. To evaluate the anti-inflammatory activity of luteoloside, this study further established a lipopolysaccharide (LPS)-induced inflammatory model in *O. cuniculus* colon epithelial cells and employed enzyme-linked immunosorbent assay (ELISA) to measure changes in the levels of key inflammatory factors (such as TNF-α and IL-6) in the cell culture supernatant.

## 2. Result and Discussion

### 2.1. Single-Factor Experimental Results

**Figure 1:**
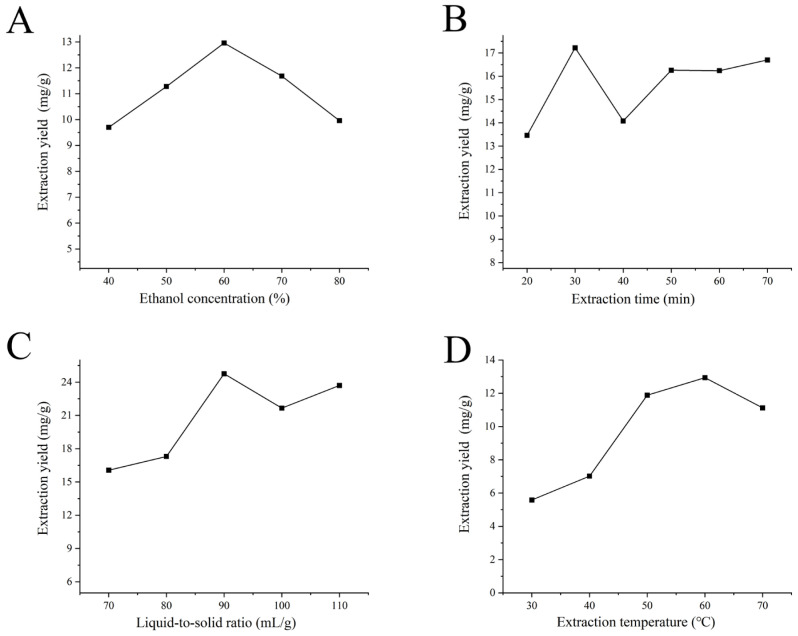
Effect of single factor on the extraction rate of total flavonoids from *S. oleraceus* in Xinjiang. (**A**) Ethanol concentration; (**B**) Extraction time; (**C**) Liquid–solid ratio; (**D**) Extraction temperature.

The efficacy of hydroalcoholic mixtures (ethanol-water) in recovering phenols and flavonoids from diverse plant matrices has been a subject of prior investigation [26]. This practice is fundamentally rooted in the principle of “like dissolves like,” which dictates that the target analyte must exhibit high solubility in the selected medium [27]. The aqueous ethanol system is particularly effective due to its enhanced solvation capacity for a wide polarity range of phenolic constituents [28]. As shown in Figure 1A, when the volume fraction of the extraction solvent (ethanol) was low, the extraction yield of total flavonoids from *S. oleraceus* was also low. When the ethanol volume fraction reached 60%, the extraction yield of total flavonoids reached its maximum value of 12.96 mg/g. Further increasing the ethanol volume fraction resulted in a decrease in extraction yield. This is because 60% ethanol solution has the closest polarity to flavonoids, making it the most suitable extraction solvent.

As shown in Figure 1B, the extraction yield of total flavonoids from *S. oleraceus* increased with longer extraction times. When the extraction time reached 30 min, the total flavonoid yield reached its maximum value of 17.22 mg/g. Continuing to extend the extraction time resulted in a decrease in the extraction yield. This is because, as the extraction time increased, the dissolution of total flavonoids from the plant material increased, peaking at 30 min. Beyond this point, further extension of the extraction time, combined with the influence of temperature, led to the decomposition of flavonoids, thereby reducing the extraction yield.

A substantial body of the literature has demonstrated that an appropriate liquid-to-solid ratio enhances the extraction yield of flavonoids [29,30]. This finding underscores why the liquid-to-solid ratio is widely recognized as an important process parameter that significantly affects overall extraction efficiency [31]. As shown in Figure 1C, when the solid-to-liquid ratio was low, the extraction yield of total flavonoids from *S. oleraceus* increased as the ratio increased. When the solid-to-liquid ratio reached 1:90, the extraction yield of total flavonoids reached its maximum value of 21.44 mg/g. When the solid-to-liquid ratio exceeded 1:90, the extraction yield decreased. This is because, as the extraction solution volume increased, flavonoids were sufficiently dissolved, reaching the highest yield at 1:90. Beyond this point, further increases in the extraction solution volume led to greater experimental errors, while also considering solvent consumption and energy loss. Therefore, a solid-to-liquid ratio of 1:90 was determined to be the most suitable.

Higher extraction temperatures intensify molecular movement, which in turn improves mass transfer and facilitates the rapid dissolution of analytes [32]. As shown in Figure 1D, the extraction yield of total flavonoids from *S. oleraceus* increased with higher extraction temperatures. When the extraction temperature reached 60 °C, the total flavonoid yield reached its maximum value of 12.94 mg/g. At lower extraction temperatures, the yield was significantly reduced. When the extraction temperature was further increased beyond 60 °C, the yield also showed a declining trend. This is because, while higher temperatures accelerate molecular movement and facilitate the dissolution of flavonoids, prolonged exposure to high temperatures can partially degrade flavonoid compounds, leading to a decrease in extraction yield.

### 2.2. Analysis of Response Surface Optimization Experimental Results

#### 2.2.1. Box–Behnken Experimental Design and Results

Taking the extraction yield of total flavonoids as the response value, factor levels were coded according to the Box–Behnken design. The results are shown in Table 1.

#### 2.2.2. Analysis of Variance for the Total Flavonoids Regression Equation and Response Surface Experimental Results

Taking ethanol volume fraction, extraction time, solid-to-liquid ratio, and extraction temperature as response variables, and the extraction yield of total flavonoids as the response value, a quadratic regression multivariate equation was established as follows:

Y = 21.25 + 0.798333A − 0.0258333B + 1.13333C + 1.06917D + 0.8125AB − 1.035AC − 0.3475AD − 0.2375BC − 0.0025BD − 0.7075CD − 1.19292A^2^ − 2.25417B^2^ − 2.72792C^2^ − 1.05417D^2^

As shown in Table 2, the model exhibited a *p*-value < 0.0001 (highly significant), while the lack-of-fit term had a *p*-value of 0.5375 (not significant). The model’s R^2^ = 0.9786 and adjusted R^2^ = 0.9536, indicating that the model can fit the experimental data with 97% accuracy. This suggests that the model can be reliably used to determine the optimal extraction process for total flavonoids from Xinjiang *S. oleraceus*. The first-order terms of ethanol concentration, solid-to-liquid ratio, and extraction temperature all reached highly significant levels (*p* < 0.01), indicating that these three factors have a pronounced linear effect on the extraction yield of total flavonoids. The four quadratic terms also showed significant curved surface effects on the extraction yield. Additionally, the interaction terms AB, AC, and CD had highly significant effects on the extraction yield (*p* < 0.01), suggesting that the influence of these factors on the extraction yield is not a simple linear relationship.

Analysis of the single-factor effects revealed that ethanol concentration, liquid-to-solid ratio, and extraction temperature all exhibited highly significant influences on the extraction yield (*p* < 0.01). With increasing ethanol concentration, the extraction yield showed a trend of first increasing and then decreasing. This phenomenon might be attributed to the fact that an appropriate hydroalcoholic ratio favors the solubility of target compounds; however, excessively high ethanol concentration alters the polarity of the solvent, thereby inhibiting the dissolution of target compounds while potentially increasing the leaching of alcohol-soluble impurities. Similarly, an appropriate increase in extraction temperature initially accelerates molecular movement and mass transfer processes, yet excessively high temperatures may lead to the degradation or structural alteration of certain heat-sensitive components, resulting in a decline in extraction yield.

**Figure 2:**
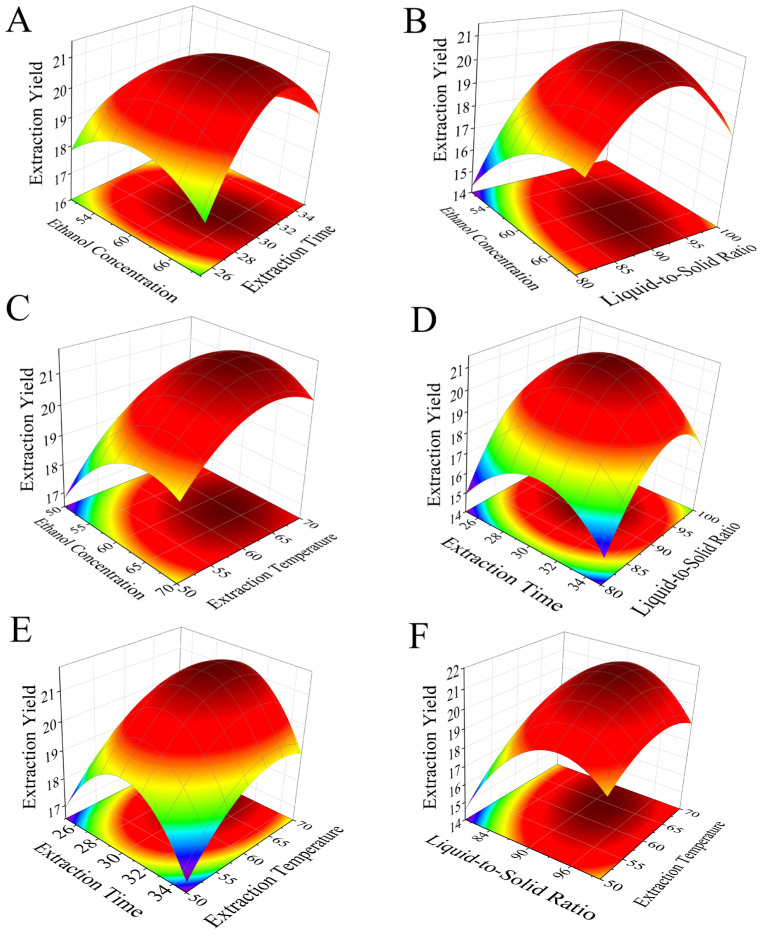
Response surface diagram of the interaction of various factors affecting the extraction rate of total flavonoids. (**A**) Ethanol concentration–extraction time; (**B**) ethanol concentration–liquid-to-solid ratio; (**C**) ethanol concentration–extraction temperature; (**D**) extraction time–liquid-to-solid ratio; (**E**) extraction time–extraction temperature; (**F**) liquid-to-solid ratio–extraction temperature. The redder the color of the response surface in the figure, the higher the extraction yield.

The response surface plots illustrating the interactive effects of various factors on flavonoid extraction yield are shown in Figure 2. The steepness of the response surface curves visually reflects the influence of each factor on the response value. There are interactions between each pair of factors, with particularly strong interactions observed between ethanol concentration and extraction time, ethanol concentration and solid-to-liquid ratio, and solid-to-liquid ratio and extraction temperature, as evidenced by the steeper changes in their corresponding response surfaces. In contrast, the interaction between extraction time and extraction temperature is the weakest, reflected by a relatively gentle response surface.

By solving the regression equation to maximize the total flavonoid extraction yield, the optimal conditions were determined as follows: ethanol concentration 62.36%, extraction time 30.15 min, solid-to-liquid ratio 1: 91.05 g/mL, and extraction temperature 64.33 °C. Under these conditions, the theoretical maximum flavonoid extraction yield is 21.64 mg/g. Considering practical laboratory constraints, the final optimized extraction conditions were adjusted to: ethanol concentration 62%, extraction time 30 min, solid-to-liquid ratio 1: 91 g/mL, and extraction temperature 64 °C. Under these adjusted conditions, three parallel experiments were conducted, yielding an average flavonoid extraction of 21.56 mg/g.

### 2.3. Determination of Luteoloside Content from S. oleraceus in Xinjiang

**Figure 3:**
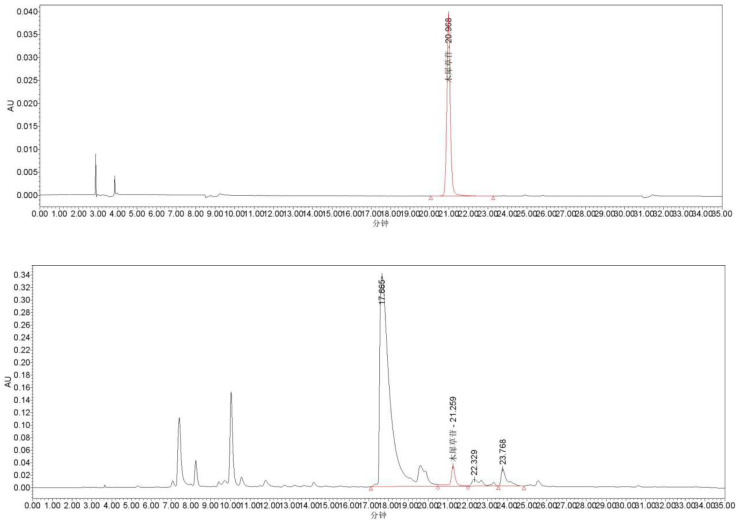
Luteoloside standard (**upper**) and sample liquid phase diagram (**lower**). The Chinese characters “木犀草苷” in the figure represent “luteoloside”, and “分钟” represents the unit of time “min”. The various shapes and red lines in the figure have no practical significance.

The content of the purified luteoloside monomer was determined using HPLC. Figure 3 (upper) shows the HPLC chromatogram of the luteoloside standard, and Figure 3 (lower) displays the chromatogram of the purified ethanol extract (x-axis: time; y-axis: absorbance). HPLC conditions: A C_18_ column was used as the stationary phase, with a detection wavelength of 350 nm. The mobile phase consisted of acetonitrile (A) and 0.5% glacial acetic acid aqueous solution (B), with a gradient program as follows: 0–30 min: 10–30% A (*v*/*v*), 90–70% B (*v*/*v*).

As shown in Figure 3, luteoloside eluted at approximately 21 min, with a peak area of 520,899 mV·s. Based on calculations, the content of luteoloside purified via polyamide column chromatography was determined to be 44.06 μg/g.

### 2.4. Effects of Different Concentrations of Luteoloside on the Viability of O. cuniculus Colon Epithelial Cells

**Figure 4:**
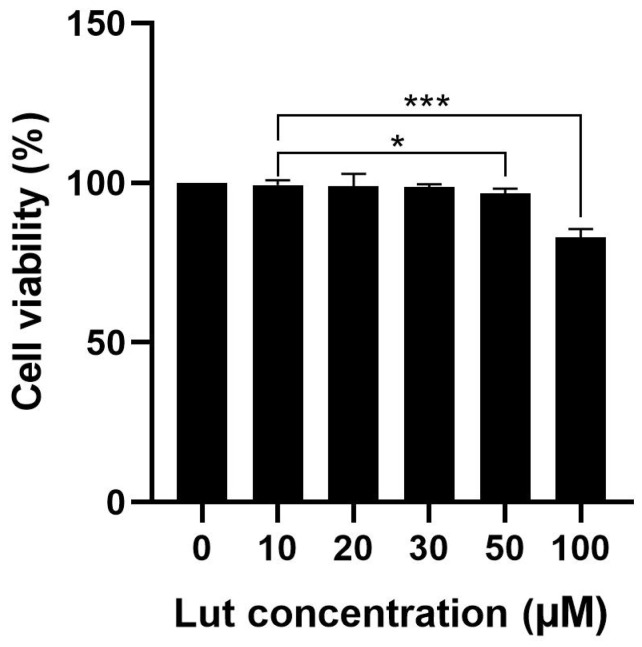
Effects of different concentrations of luteoloside on the viability of *O. cuniculus* colon epithelial cells. * *p* < 0.05, *** *p* < 0.001.

To evaluate the potential toxic effects of luteoloside on *O. cuniculus* colon epithelial cells and determine suitable concentrations for subsequent experiments, this study employed the CCK-8 assay to measure cell viability after 24 h of treatment with varying concentrations of luteoloside. As shown in Figure 4, the effect of luteoloside on cell viability exhibited a clear concentration-dependent pattern. At concentrations below 50 µmol/L, cell viability showed no significant difference compared to the blank control group, consistently remaining above 95%. This result indicates that within this concentration range, luteoloside has no obvious toxic effect on *O. cuniculus* colon epithelial cells, and the cells maintained good growth conditions, suggesting a high level of biosafety. However, when the concentration increased to 100 µmol/L, a significant decline in cell viability was observed, dropping to approximately 80%. This suggests that at higher concentrations, luteoloside begins to exhibit mild inhibitory effects on cell proliferation or induces some level of cytotoxicity. To ensure that subsequent experiments could effectively observe the bioactive effects of luteoloside while reasonably assessing its potential cellular stress responses, this study ultimately selected concentrations of 50 µmol/L and 100 µmol/L luteoloside for further experimental exploration.

### 2.5. Protective Effect of Luteoloside on LPS-Induced Cellular Damage

**Figure 5:**
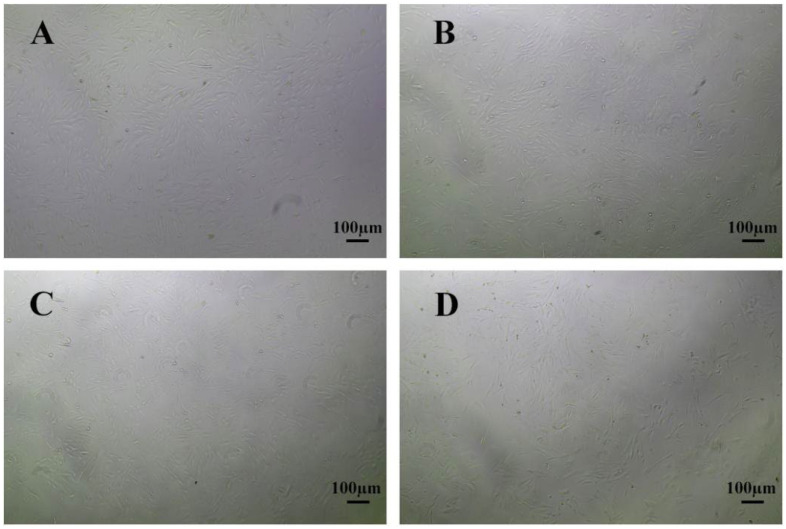
Effects of LPS and luteoloside on the viability of *O. cuniculus* colon epithelial cells. (**A**) *O. cuniculus* colon epithelial cells; (**B**) *O. cuniculus* colon epithelial cells were treated with 1 μg/mL LPS; (**C**) *O. cuniculus* colon epithelial cells were treated with LPS and 50 μmol/L luteoloside at the same time; (**D**) *O. cuniculus* colon epithelial cells were treated with LPS and 100 μmol/L luteoloside at the same time.

**Figure 6:**
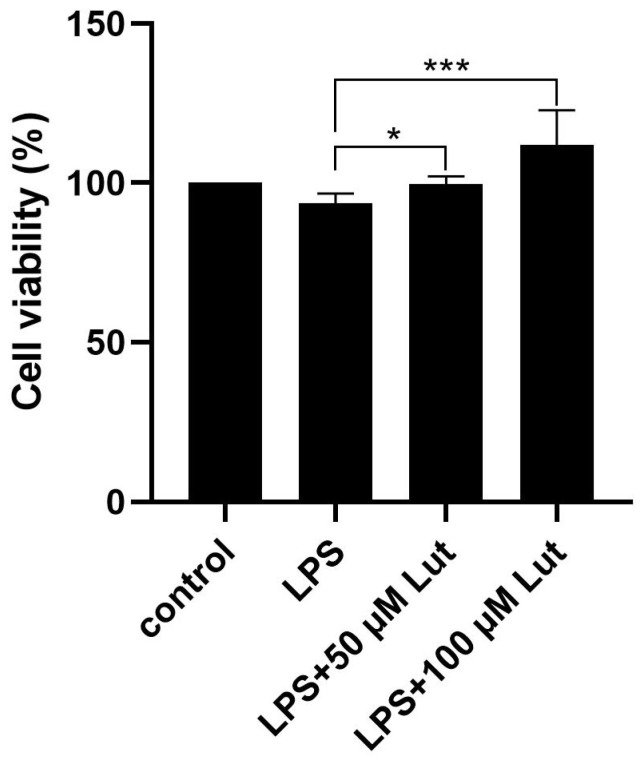
Detection of cell viability by CCK-8. * *p* < 0.05, *** *p* < 0.001.

To evaluate the protective effect of luteoloside on lipopolysaccharide (LPS)-induced injury in *O. cuniculus* colon epithelial cells, we assessed cell viability using the CCK-8 assay. As shown in Figure 5 and Figure 6, compared with the normal control group, LPS treatment alone caused a highly significant decrease in cell viability (*p* < 0.01), indicating successful establishment of the cellular inflammatory injury model. However, compared with the LPS model group, pretreatment with 50 µmol/L and 100 µmol/L luteoloside significantly restored cell viability, demonstrating that luteoloside effectively reversed LPS-induced cytotoxicity and markedly improved cell survival under inflammatory stress.

### 2.6. Determination of TNF-α, IL-1β, IL-6 and IL-10 Levels in Cell Supernatants by ELISA

**Figure 7:**
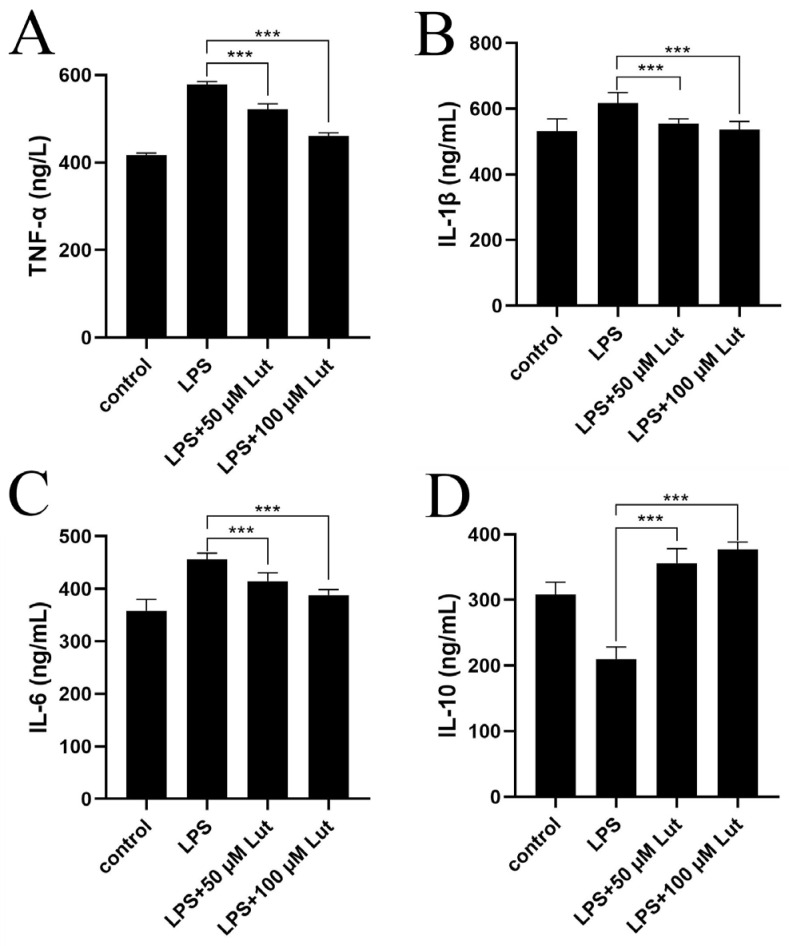
The contents of TNF-α, IL-1β, IL-6 and IL-10 in the supernatant of cells were detected by ELISA. (**A**) TNF-α; (**B**) IL-1β; (**C**) IL-6; (**D**) IL-10. *** *p* < 0.001.

As shown in Figure 7, compared with the normal control group, the expression levels of pro-inflammatory factors IL-1β and IL-6 in LPS-treated cells were significantly increased (Figure 7A–C), indicating that LPS successfully induced an inflammatory response in intestinal epithelial cells and that the intestinal inflammation cell model was successfully established. On the basis of LPS induction, 50 µmol/L and 100 µmol/L luteoloside were added respectively for intervention. The results showed that, compared with the LPS-only treatment group, the combined luteoloside treatment groups exhibited significantly reduced expression of the pro-inflammatory factors TNF-α, IL-1β and IL-6, with a certain dose-dependent trend. At the same time, the expression of the anti-inflammatory factor IL-10 was significantly increased (Figure 7D). These results indicate that luteoloside can effectively inhibit the release of LPS-induced pro-inflammatory cytokines (TNF-α, IL-1β, IL-6) and promote the expression of the anti-inflammatory factor IL-10. This effect suggests that luteoloside has a pronounced anti-inflammatory activity against LPS-induced inflammation in intestinal epithelial cells, likely by modulating the balance of inflammatory mediators and alleviating the cellular inflammatory state.

An interesting observation was that treatment with 100 μmol/L luteoloside alone significantly reduced the viability of *O. cuniculus* colon epithelial cells, suggesting a potential cytotoxic effect at this concentration. However, when cells were co-treated with luteoloside and LPS, cell viability was not only restored but appeared to be enhanced compared to cells treated with LPS alone. This phenomenon could be attributed to the dual role of flavonoids at high concentrations. It is plausible that in the absence of stress (LPS-untreated condition), the high dose of luteoloside exerts mild cytotoxic effects, possibly through pro-oxidant activities. Conversely, under inflammatory conditions induced by LPS, the strong anti-inflammatory properties of luteoloside—such as the inhibition of TNF-α, IL-1β, and IL-6—may alleviate LPS-induced cellular damage and metabolic disruption, thereby preserving cell viability. This suggests that the protective effects of luteoloside become predominant in a pathological context, highlighting its potential as a therapeutic agent for inflammation-related intestinal damage. Further studies are needed to elucidate the precise molecular switch governing this context-dependent effect.

Luteoloside, a naturally occurring flavonoid widely present in medicinal plants such as *Lonicera japonica* (honeysuckle) and *Chrysanthemum morifolium*, has garnered widespread attention in recent years due to its notable anti-inflammatory activity. Studies have demonstrated that luteoloside exerts potent anti-inflammatory effects across various inflammatory models. In a mouse model of inflammatory pain induced by Complete Freund’s Adjuvant (CFA), luteoloside not only significantly elevated mechanical and thermal pain thresholds in a dose-dependent manner, but also effectively reduced the levels of the pro-inflammatory cytokine interleukin-1β (IL-1β) in the plantar tissue, dorsal root ganglia (DRG), serum, and spinal dorsal horn [18].

Mechanistically, current research has primarily elucidated the following aspects: luteoloside exerts anti-inflammatory and anti-osteoclastogenic effects by blocking NFATc1 activity and attenuating RANKL-mediated Ca^2+^ signaling [33]; it inhibits the activation of macrophages in the DRG and microglia in the spinal dorsal horn, thereby reducing the production of inflammatory mediators at their source [34]; and drawing on findings related to its aglycone, luteolin, luteoloside may exert synergistic anti-inflammatory effects through the modulation of multiple signaling pathways, including NF-κB [35], JAK/STAT [36], and Nrf2/HO-1 [37].

Notably, as the 7-O-glucoside of luteolin, the glycosylation of luteoloside enhances its water solubility, which may influence its in vivo absorption and distribution. Further comparative pharmacological studies are warranted to elucidate the differences in anti-inflammatory activity between luteolin and luteoloside, as well as the underlying mechanisms. In summary, luteoloside exerts its anti-inflammatory effects through multiple pathways, including the inhibition of pro-inflammatory cytokine production, regulation of MEKK3 expression, and suppression of immune cell activation, highlighting its potential therapeutic value in the treatment of inflammatory diseases.

## 3. Materials and Methods

### 3.1. Experimental Materials

The experimental material was *S. oleraceus* collected from the surrounding areas of the Tarim Basin in Xinjiang. Standards of rutin and luteoloside were used. The reagents included CCK-8 kit. ELISA kits were purchased from Enzyme Immunity Industry Co., Ltd., Yancheng, China (Cat. No.: MM-0240O1, MM-0305O1, MM-0302O1, MM-0307O1). D101 macroporous resin, polyamide powder, aluminum nitrate nonahydrate, sodium hydroxide, sodium nitrite, absolute ethanol, and *O. cuniculus* colonic epithelial cells were obtained from Fenghui Biotechnology Co., Ltd., Changsha, China (Cat. No. FHRAB707). LPS was purchased from MedChemExpress LLC, Monmouth Junction, NJ, USA (Cat. No. HY-D1056).

### 3.2. Experimental Instruments

High-speed pulverizer (WK-1400A, Jingcheng Pharmaceutical Equipment Manufacturing Co., Ltd., Zibo, China), ultrasonic cleaner (KQ5200DE, Kunshan Ultrasonic Instruments Co., Ltd., Kunshan, China), oil-free vacuum pump (LC-VP-85, Lichen-BX instrument Technology Co., Ltd., Shanghai, China), microplate reader (K6600A+, Kaiao Technology Development Co., Ltd., Beijing, China), rotary evaporator (SN-RE-1002, Shangpu Instrument Equipment Co., Ltd., Shanghai, China), and high-performance liquid chromatography (HPLC) system (Waters Corporation, Milford, MA, USA).

### 3.3. Experimental Methods

#### 3.3.1. Preparation of *S. oleraceus* Powder

The collected *S. oleraceus* was washed, air-dried in the shade, and pulverized using a high-speed pulverizer. The dried material was sieved through an 80-mesh standard sieve to obtain a fine powder, which was then stored in a refrigerator at 4 °C until use.

#### 3.3.2. Extraction of Flavonoids from *S. oleraceus*

Two grams (2.0 g) of *S. oleraceus* powder was accurately weighed using an electronic analytical balance (precision: 0.0001 g) and transferred into a 250 mL conical flask. A specified volume of ethanol extraction solvent was added to the flask at a pre-defined solid-to-liquid ratio (*w*/*v*), and the mixture was allowed to stand at room temperature for 15 min to facilitate sufficient infiltration of the solvent into the plant powder. Subsequently, ultrasound-assisted extraction was conducted under pre-set conditions, including a controlled extraction temperature, fixed extraction duration, and constant ultrasonic power. After the extraction process was completed, the mixture was immediately subjected to reduced-pressure filtration using a Buchner funnel equipped with filter paper to separate the solid residue from the liquid extract. The collected filtrate was designated as the crude flavonoid extract and stored at 4 °C in the dark until subsequent experimental analysis.

#### 3.3.3. Determination of Total Flavonoid Content

Volume of 4.0 mL of the rutin standard solution (200 mg/L) was precisely pipetted using a volumetric pipette, and full-wavelength scanning was performed over the range of 200–400 nm with 60% ethanol serving as the blank reference. The scanning results showed that the solution exhibited a maximum absorbance peak at a wavelength of 510 nm, thus confirming 510 nm as the optimal detection wavelength for the subsequent determination of total flavonoid content.

Aliquots of 0, 1, 2, 3, 4, 5, and 6 mL of the rutin standard solution were accurately pipetted into separate 25 mL volumetric flasks, respectively. Each flask was supplemented with 60% ethanol solution to a constant volume of 12.5 mL. Subsequently, 0.7 mL of 5% sodium nitrite solution was added to each flask, and the mixtures were vortexed thoroughly before being allowed to stand at room temperature for 5 min. Next, 0.7 mL of 10% aluminum nitrate solution was incorporated into the systems; the resulting solutions were mixed well and incubated for another 6 min. Thereafter, 5.0 mL of 4% sodium hydroxide solution was added to each flask. The volume of each mixture was adjusted to the 25 mL mark with 60% ethanol, followed by thorough mixing and a final standing period of 10 min. Finally, using 60% ethanol as the blank reference, the absorbance value of each solution was measured at the optimal wavelength of 510 nm.

A 10.0 mL aliquot of the crude flavonoid extract was accurately measured, diluted to 100 mL with distilled water, and mixed thoroughly. A 5.0 mL volume of the resulting diluted sample solution was then precisely transferred, and the subsequent procedures were performed in accordance with the method described for standard curve preparation. The absorbance of the sample solution was measured at 510 nm, and the corresponding rutin mass was determined from the pre-established standard curve. The flavonoid extraction yield of the sample was finally calculated using the corresponding formula.

Flavonoid extraction yield from *S. oleraceus* (mg/g) = (*C* × *N* × *V_t_*)/*m*

The total flavonoid extraction yield of *S. oleraceus* was calculated using the above formula, where the definitions of each parameter are specified as follows:

*C*: Total flavonoid concentration in the test solution, derived from the pre-established standard curve (mg/mL).

*N*: Dilution factor of the crude flavonoid extract during sample pretreatment.

*V_t_*: Total volume of the initial crude flavonoid extraction solution (mL).

*m*: Mass of the *S. oleraceus* powder used for extraction (g).

#### 3.3.4. Single-Factor Experimental Design

The effects of four key parameters—ethanol concentration, extraction time, solid-to-liquid ratio, and extraction temperature—on the extraction of flavonoids from *S. oleraceus* were systematically investigated using single-factor experiments:

Ethanol concentration (40%, 50%, 60%, 70%, 80%) was evaluated at an extraction time of 60 min, solid-to-liquid ratio of 1:30 g/mL, and extraction temperature of 50 °C.

Extraction time (20, 30, 40, 50, 60 min) was examined at 60% ethanol, 1:30 g/mL solid-to-liquid ratio, and 50 °C.

Solid-to-liquid ratio (1:60, 1:70, 1:80, 1:90, 1:100 g/mL) was studied at 60% ethanol, 60 min extraction time, and 50 °C.

Extraction temperature (30, 40, 50, 60, 70 °C) was analyzed at 60% ethanol, 60 min extraction time, and 1:30 g/mL solid-to-liquid ratio.

#### 3.3.5. Response Surface Optimization of Flavonoids Extraction from *S. oleraceus*

Based on the results of the single-factor experiments, four factors—ethanol concentration, extraction time, solid-to-liquid ratio, and extraction temperature—were selected for further optimization. Using a Box–Behnken experimental design, the factors and levels were determined, and response surface analysis was conducted with the total flavonoid extraction yield as the indicator to identify the optimal extraction conditions, as shown in Table 3.

#### 3.3.6. Purification of Flavonoids

First, 300 g of D101 macroporous adsorption resin was transferred into a large beaker, and 95% ethanol was added to the beaker until the liquid surface reached approximately 3 cm above the resin layer. The mixture was stirred gently and then left to stand undisturbed for 24 h to fully activate the resin. After the standing period, the ethanol solution was filtered out with medical gauze, and the resin was rinsed repeatedly with distilled water until no residual ethanol was detected.

Subsequently, the pre-treated resin was soaked in 2% sodium hydroxide solution for 12 h, then rinsed continuously with distilled water until the eluate reached a neutral pH. Following this, the resin was immersed in 5% dilute hydrochloric acid solution for another 12 h, and rinsed again with distilled water until the pH of the rinse solution returned to neutral. Finally, the purified resin was immersed in distilled water for subsequent experimental use, and column packing was carried out after a 2-h immersion period.

For column packing, the pre-cleaned resin column was pre-wetted with deionized water, and a small volume of deionized water was retained inside the column. A piece of degreasing cotton roughly the size of a fingernail was moistened, squeezed gently to expel any trapped air bubbles, and then placed at the bottom of the column. The column valve was opened to allow the cotton to settle steadily at the column bottom with the flow of water, after which the outlet was closed immediately to prevent potential resin leakage during subsequent operations.

Next, deionized water equivalent to 1/4 of the total column volume was added into the column. The pre-treated macroporous resin was stirred thoroughly to form a uniform suspension, which was then slowly poured into the pre-prepared column. The column valve was not opened immediately; instead, the system was left to stand undisturbed for 2 h to facilitate natural sedimentation of the resin. Any air bubbles formed within the column during this process were carefully removed manually. Once all bubbles were eliminated, the valve was opened, and the resin column was flushed with five column volumes of deionized water to compact the resin bed tightly. The liquid level was maintained at approximately 2 cm above the resin bed throughout the process, and the packed column was set aside for subsequent experiments.

The supernatant of the previously prepared crude extract was carefully withdrawn using a rubber dropper and slowly dripped along the inner wall of the resin column. Deionized water was then added to facilitate the entry of the crude extract into the column, and a distinct yellowing of the resin column was observed. The column was washed with water until the eluent exhibited a very faint color. Subsequently, sequential elution was carried out using ethanol solutions at concentrations of 10%, 20%, 30%, 40%, and 50%, respectively. The eluates obtained with 30% and 40% ethanol were combined and concentrated to a paste by rotary evaporation.

#### 3.3.7. Separation and Purification of Luteoloside

**Figure 8:**
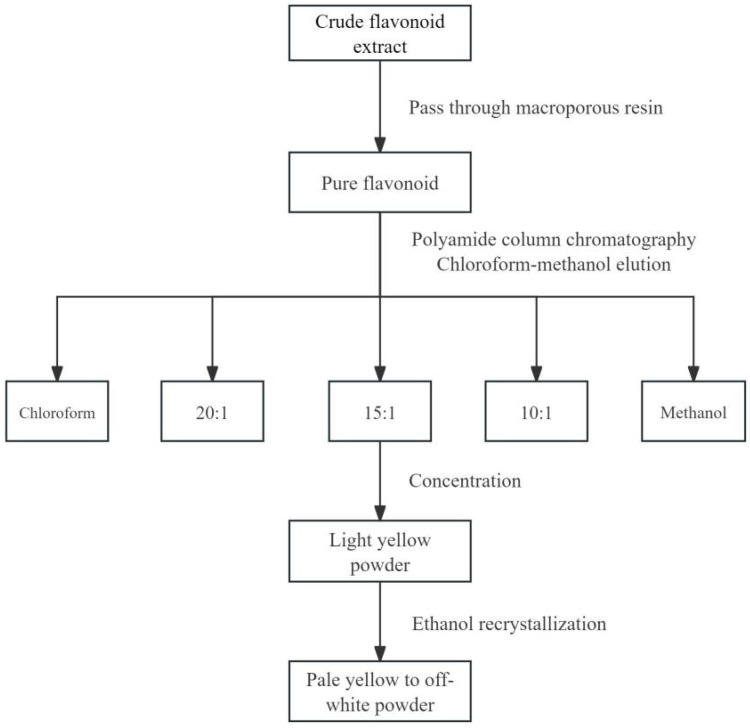
Separation and purification process of luteoloside.

As shown in Figure 8, after obtaining purified flavonoids, they were eluted using different ratios of chloroform–methanol through a polyamide column. The eluate was then concentrated and recrystallized to obtain the purified luteoloside monomer.

#### 3.3.8. Effect of Luteoloside on the Activity of *O. cuniculus* Colonic Epithelial Cells

When *O. cuniculus* colonic epithelial cells reached approximately 95% confluence in a 50 mm culture dish, 0.25% trypsin was added to digest the cells. After observing about one-third of the cells detaching, the digestion was immediately terminated by adding triple the volume of DMEM/F12 medium. The bottom of the dish was gently pipetted to collect the cells, and the cell suspension was transferred to a 15 mL centrifuge tube and centrifuged at 1000 rpm for 5 min. The supernatant was discarded, and the cells were resuspended in 10 mL of complete medium. Subsequently, 100 µL of the cell suspension was seeded into each well of a 96-well plate and cultured at 37 °C with 5% CO_2_ for 24 h.

The medium in the wells was discarded, and luteoloside stock solution diluted in DMEM/F12 medium was added at concentrations of 0, 10, 20, 30, 50, and 100 µmol/L. Each group was set up with five replicates, followed by incubation at 37 °C with 5% CO_2_ for 24 h.

The drug-containing medium was removed, and CCK-8 solution was added to each well, followed by incubation at 37 °C for 2 h. Absorbance was measured at 450 nm using a microplate reader.

Cell viability (%) = [(OD experimental group − OD blank group)/(OD negative control group − OD blank group)] × 100%

#### 3.3.9. Inhibitory Effect of Luteoloside on LPS-Induced Inflammation in Colonic Epithelial Cells

The cells were stimulated with 1 μg/mL lipopolysaccharide (LPS) from *Escherichia coli* O55:B5 for 24 h. The method was the same as in Section 3.3.8 for seeding the 96-well plate. The experiment was divided into four main groups: Control group, LPS group, LPS + 50 µmol/L luteoloside group, and LPS + 100 µmol/L luteoloside group. After 24 h, cell activity was detected using the CCK-8 assay. Following the 24-h incubation, cell culture medium supernatant was collected and centrifuged at 3000 rpm for 10 min to remove particles and polymers. The concentrations of TNF-α, IL-1β, IL-6, and IL-10 in the cells were then determined according to the ELISA kit instructions.

### 3.4. Statistical Analysis

All experiments were performed in triplicate, and data were expressed as the mean ± standard deviation (SD). Statistical analysis was carried out using IBM SPSS Statistics Grad Pack (v30.0.0.0) software. One-way analysis of variance (ANOVA) was applied to evaluate significant differences among groups. Differences were considered statistically significant at *p* < 0.05 and highly significant at *p* < 0.01.

## 4. Conclusions

In this study, a response surface methodology was employed to optimize the extraction of flavonoids from *S. oleraceus*. The optimal extraction conditions were determined as follows: ethanol concentration of 62%, extraction time of 30 min, liquid-to-solid ratio of 1:91 g/mL, and extraction temperature of 64 °C. Under these conditions, the total flavonoid yield reached 21.56 mg/g. Subsequently, luteoloside was isolated and purified using polyamide column chromatography, and its content was determined by HPLC to be 44.06 μg/g. In vitro cellular assays demonstrated that luteoloside exerts significant anti-inflammatory effects in LPS-induced colon epithelial cells of *O*. *cuniculus* by inhibiting the release of pro-inflammatory cytokines (TNF-α, IL-1β, and IL-6) while promoting the expression of the anti-inflammatory cytokine IL-10. These findings not only establish an efficient protocol for the extraction and purification of luteoloside from *S. oleraceus* but also provide experimental evidence for its anti-inflammatory activity at the cellular level. Future in vivo studies in *O. cuniculus* are warranted to further validate its therapeutic efficacy and pharmacokinetic profile. Given the prevalence of inflammatory diseases in the *O. cuniculus* breeding industry, luteoloside shows promise as a natural feed additive or therapeutic agent for improving animal health and promoting sustainable livestock production.

## Figures and Tables

**Table 1:** Response surface design and results of extraction rate of flavonoids from *S. oleraceus* in Xinjiang.

Experiment Number	A Ethanol Concentration (%)	B Extraction Time (min)	C Liquid-to-Solid Ratio (mL/g)	D Extraction Temperature (°C)	Extraction Yield (mg/g)
1	50	25	90	60	18.18
2	70	25	90	60	18.11
3	50	35	90	60	15.74
4	70	35	90	60	18.92
5	60	30	80	50	14.09
6	60	30	100	50	18.06
7	60	30	80	70	18.16
8	60	30	100	70	19.30
9	50	30	90	50	16.78
10	70	30	90	50	19.25
11	50	30	90	70	19.26
12	70	30	90	70	20.34
13	60	25	80	60	14.80
14	60	35	80	60	15.47
15	60	25	100	60	17.35
16	60	35	100	60	17.07
17	50	30	80	60	14.64
18	70	30	80	60	18.17
19	50	30	100	60	18.88
20	70	30	100	60	18.27
21	60	25	90	50	16.88
22	60	35	90	50	17.35
23	60	25	90	70	18.86
24	60	35	90	70	19.32
25	60	30	90	60	20.80
26	60	30	90	60	21.46
27	60	30	90	60	21.49

**Table 2:** Analysis of variance of total flavonoids extraction response surface model.

Source	Sum of Squares	df	Mean Square	F-Value	*p*-Value	
Model	97.20	14	6.94	39.14	<0.0001	**
A	7.65	1	7.65	43.12	<0.0001	**
B	0.0080	1	0.0080	0.0451	0.8353	
C	15.41	1	15.41	86.89	<0.0001	**
D	13.72	1	13.72	77.33	<0.0001	**
AB	2.64	1	2.64	14.89	0.0023	**
AC	4.28	1	4.28	24.16	0.0004	**
AD	0.4830	1	0.4830	2.72	0.1248	
BC	0.2256	1	0.2256	1.27	0.2814	
BD	0.0000	1	0.0000	0.0001	0.9907	
CD	2.00	1	2.00	11.29	0.0057	**
A^2^	7.59	1	7.59	42.79	<0.0001	**
B^2^	27.10	1	27.10	152.78	<0.0001	**
C^2^	39.69	1	39.69	223.74	<0.0001	**
D^2^	5.93	1	5.93	33.41	<0.0001	**
Residual	2.13	12	0.1774			
Lack of Fit	1.82	10	0.1824	1.20	0.5375	
Pure Error	0.3042	2	0.1521			
Cor Total	99.33	26				
R^2^	0.9786					
Adjusted R^2^	0.9536					
Predicted R^2^	0.8873					

In the table, “**” indicates a highly significant difference.

**Table 3:** Box–Behnken experimental design factors and levels.

Factors	Levels		
Ethanol concentration (%)	50	60	70
Extraction time (min)	25	30	35
Liquid-to-solid ratio (mL/g)	80	90	100
Extraction temperature (°C)	50	60	70

## Data Availability

The data presented in this study are available on request from the corresponding author.
